# Bimaxillary Aneurismal Bone Cyst in Patient with End Stage Renal Disease and Hyperparathyroidism: A Rare Case Report and Review of the Literature

**DOI:** 10.1155/2016/7026106

**Published:** 2016-10-05

**Authors:** Sedigheh Bakhtiari, Mahin Bakhshi, Fatemeh Mashhadiabbas, Hasan Mir Mohammad Sadeghi, Zahra Elmi Rankohi, Somayeh Rahmani

**Affiliations:** ^1^Department of Oral Medicine, School of Dentistry, Shahid Beheshti University of Medical Sciences, Tehran, Iran; ^2^Department of Oral & Maxillofacial Pathology, School of Dentistry, Shahid Beheshti University of Medical Sciences, Tehran, Iran; ^3^Department of Oral & Maxillofacial Surgery, School of Dentistry, Shahid Beheshti University of Medical Sciences, Tehran, Iran; ^4^Oral Medicine Department, Dental School, Gilan University of Medical Sciences, Rasht, Iran

## Abstract

Aneurismal bone cyst (ABC) is a rare bony lesion occurring predominantly in long bones. Its jaws' involvement is uncommon and the simultaneous involvement of both jaws is very rare. This report is about a 27-year-old female experiencing renal failure with ABC involving her maxilla and mandible. The progressive lesion was treated surgically and there was no recurrence after 18 months of follow-up.

## 1. Introduction

The first clinicopathological description of aneurismal bone cyst (ABC) was done by Jaffe and Lichtenstein in 1942 [1]. ABC is not lined by epithelium and it is not a true cyst. According to the definition of the World Health Organization (WHO), ABC is a benign tumor-like lesion [2]. The term “aneurismal” was used to refer to this lesion because of the “blow-out” effect or affected bone that appears in this type of lesion [3, 4]. The most common locations of ABCs are long bones and the vertebral column. The frequency of skull and mandibular occurrence is 4% [5]. Jaws involvement is uncommon and only 1.9–2% has been reported [1, 6].

ABC has extreme variable clinical presentations; the most typical feature is the well-defined swelling of soft tissue when it increases the size of the adjacent bone [3]. As usual, ABC has a slow growing painless mass until erosion of the cortical plates occur, thereby showing a rapid growth which may cause pain. Other clinical presentations are facial deformity, malocclusion, tooth mobility, neurologic symptoms including dysesthesias and paresthesia, progressive nasal obstruction due to sinus involvement of the maxillary lesions, ptosis, diplopia, pathologic fracture, perforation of cortex, and hearing of bruit if the arterial component is involved significantly [3, 7–9].

Radiographic examination shows a unilocular or multilocular radiolucency, often with marked cortical expansion and thinning and the border of lesion may be well defined or poorly defined. Ballooning or blow-out distention of the affected bone may be seen.

A frequent histologic feature of ABC is the vascular form with blood-filled cavernous and sinusoid spaces with a variable amount of hemosiderin, pigmentation, and giant cells. There are evidences of reactive and woven bone in connective tissues, as well [3, 4, 6].

In Kalantar Motamedi et al.'s study, from 120 cases of maxillofacial ABC, only one case had concurrent mandibular and maxillary ABC [7]. We reported a rare case of bimaxillary ABC in a patient with End Stage Renal Disease (ESRD) and hyperparathyroidism.

## 2. Case Presentation

**Figure 1:**
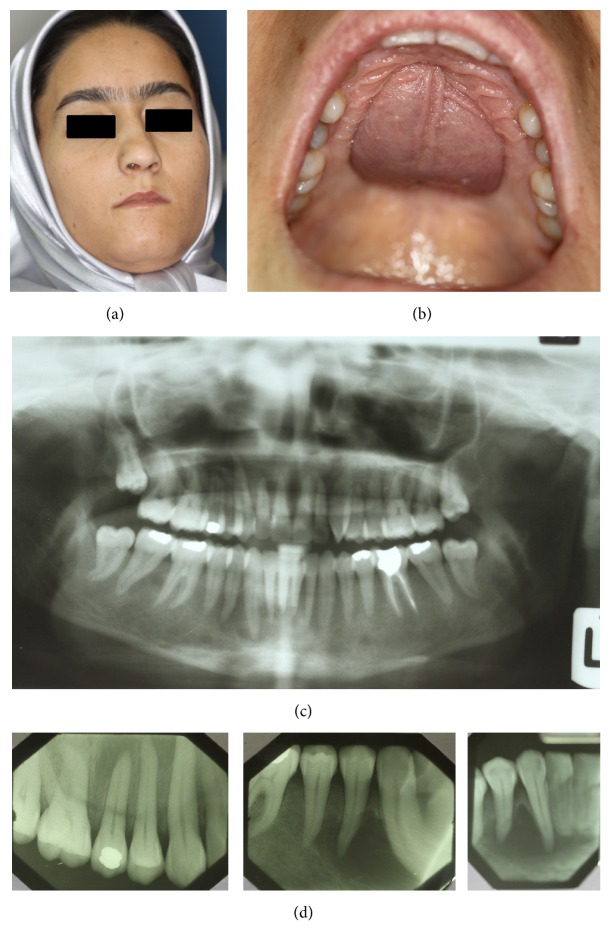
First visit: (a) extraoral frontal photography, (b) intraoral photography, and (c) panoramic and (d) periapical views.

On May 2013, a 27-year-old female was referred to an Oral Medicine Department in Tehran, Iran, with the chief complaint of a mass in her palate (Figures 1(a) and 1(b)). Initially, she complained about the lesion a week prior to the referral. The patient's medical history revealed that she had End Stage Renal Disease (ESRD) and received hemodialysis treatment three times a week for the past 11 years; she also had hypertension. A parathyroid scan revealed parathyroid adenoma in the lower portion of the left thyroid lobe.

Intraoral examination showed an exophytic dome-shaped lesion with smooth surface in the midportion of the hard palate with an approximate size of 40 × 50 mm. The color of the lesion was pink to purple with firm-to-hard consistency and nontenderness in palpation (Figure 1(b)).

In panoramic view, bony rarefaction was evidenced in the right quadrant of maxilla, in the region of canine and premolars. Also accidentally in panoramic and periapical radiography, a well-defined unilocular radiolucent lesion with scalloped border on the right quadrant of mandible from the central incisor to second premolar was observed (Figures 1(c) and 1(d)).  Table 1 shows the results of laboratory tests.

According to patient's medical history, she was referred to her physician about controlling of hyperparathyroidism. Parathyroidectomy was done on August 2013. A whole body bone scan showed multiple foci of increased radiotracer uptake in the nasofacial bones, spine, lower third of the right humerus, bilateral ribs, pelvic bones, and both tibiae. Regarding patient history, the scan pattern favored metabolic bone disease (hyperparathyroidism and renal osteodystrophy) (Figure 3).

Six months after, the patient was referred to our clinic with swelling of her right maxilla with progressive growth and pain.

**Figure 2:**
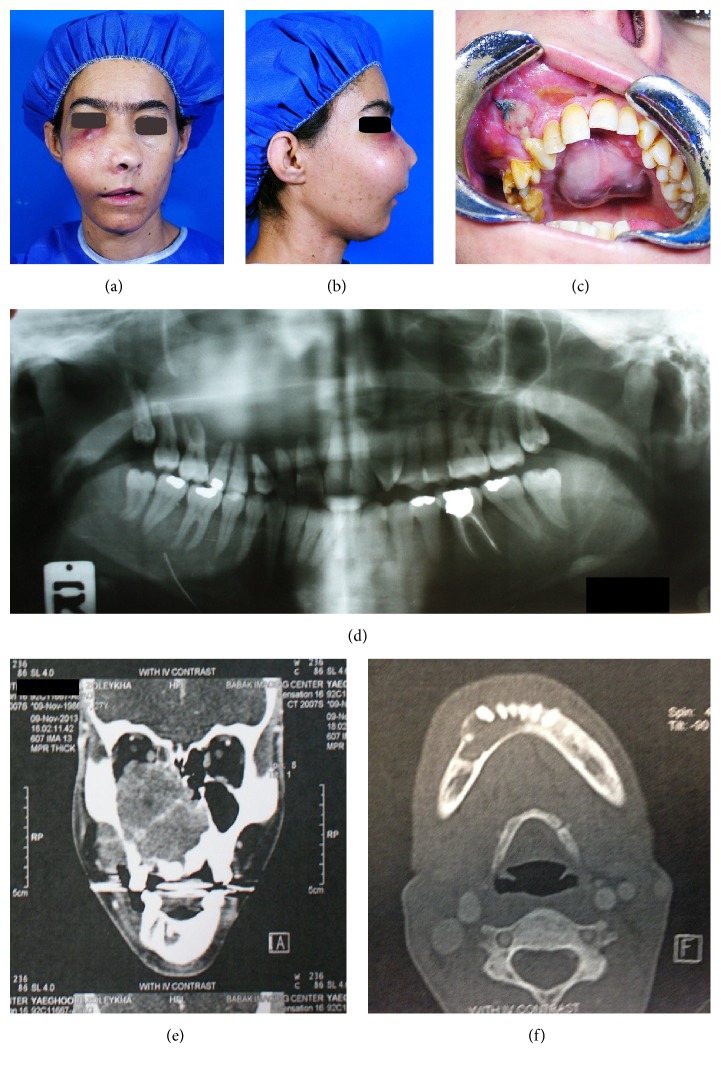
After 6 months, preoperative (a, b) extraoral frontal and lateral photography were carried out. (c) Intraoral photography. (d) Panoramic view. (e) Coronal contrast enhanced CT scan image. (f) Axial contrast enhanced CT scan image.

**Figure 3:**
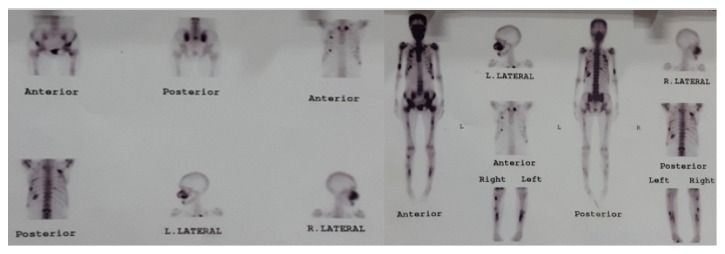
Preoperative whole body scan.

Extraoral findings showed a large mass with bony hard consistency on the right maxilla, causing nasal deformity with nasal obstruction and a nodular firm mass with purple color in the infraorbital region. Also, superior displacement of the right glob was seen (Figures 2(a) and 2(b)).

In addition to hard palate lesion intraoral findings shown, a firm mass of the right buccal sulcus and displacement and loosening of the right upper teeth were seen (Figure 2(c)).

Panoramic radiograph revealed large radiolucent lesion involving the right maxilla, and the maxillary sinus had no obvious margins. Teeth displacement was seen in the right maxilla (Figure 2(d)). Contrast enhanced CT scan of the coronal axis showed a heterogeneous mass in the right maxilla; the lesion crossed midline and invaded to medial wall of the left maxillary sinus. Also, in the buccal side, the alveolar process was destructed and a soft tissue mass was formed (Figure 2(e)).

In CT there was an osteolytic lesion in the right side of the mandibular body with extension from the midline to premolar area. Expansion and thinning of the buccal cortex, thinning of the lingual cortex, and buccal movement of teeth were seen (Figure 2(f)).

According to medical history of this case and clinical and radiological findings Brown tumor and central giant cell granuloma were suspected.

**Figure 4:**
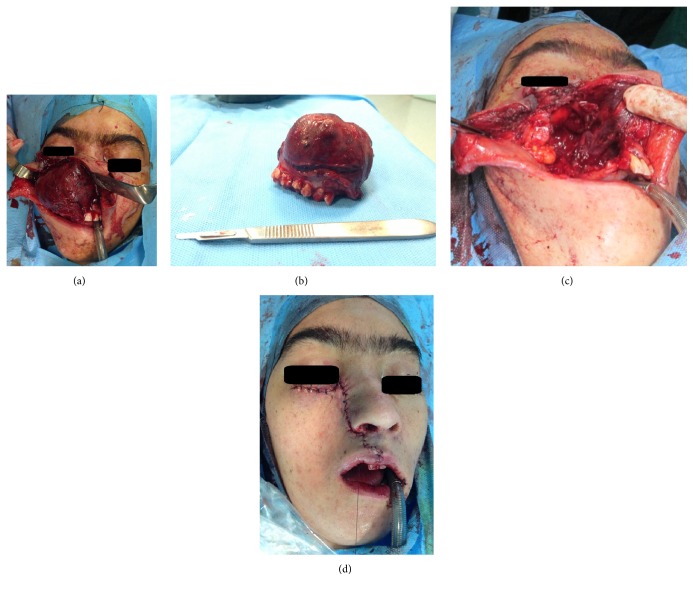
Intraoperative photograph shows the (a) macroscopic appearance of the lesion. (b, c) Resected specimen. (d) Closure.

**Figure 5:**
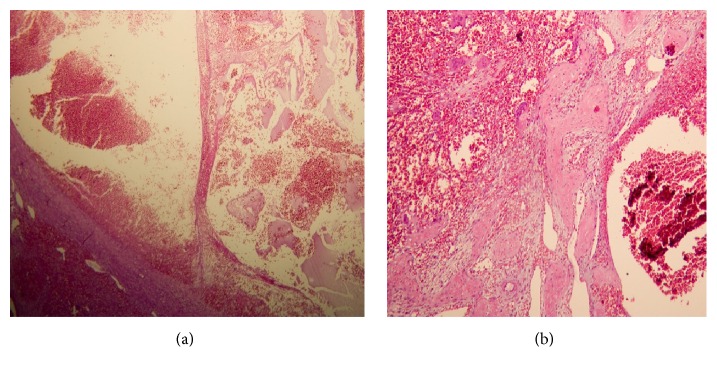
Histopathology photomicrograph. (a) Hematoxylin and eosin stained tissue sections of the mandibular lesion [HPF ×40]. (b) H&E stained tissue sections of the maxillary lesion show new bone formation, hemorrhage, giant cells, and sinusoid space in cellular fibrous connective tissue background [HPF ×100].

Patient informed consent was obtained. Excisional biopsy by hemimaxillectomy under general anesthesia and Weber-Ferguson approach was done, also in the mandible, incisional biopsy with extraction of right lower canine was done (Figure 4). The excised specimen was sent for microscopic examination. A blood-filled space with varying size without endothelial lining, surrounded by fibroblastic connective tissue, was seen. Chronic inflammatory cell infiltration and multinucleated giant cells with hemorrhage and hemosiderin pigmentation were seen. Fragments of new bone formation and osteoid were seen. The features were consistent with an ABC (Figure 5).

**Figure 6:**
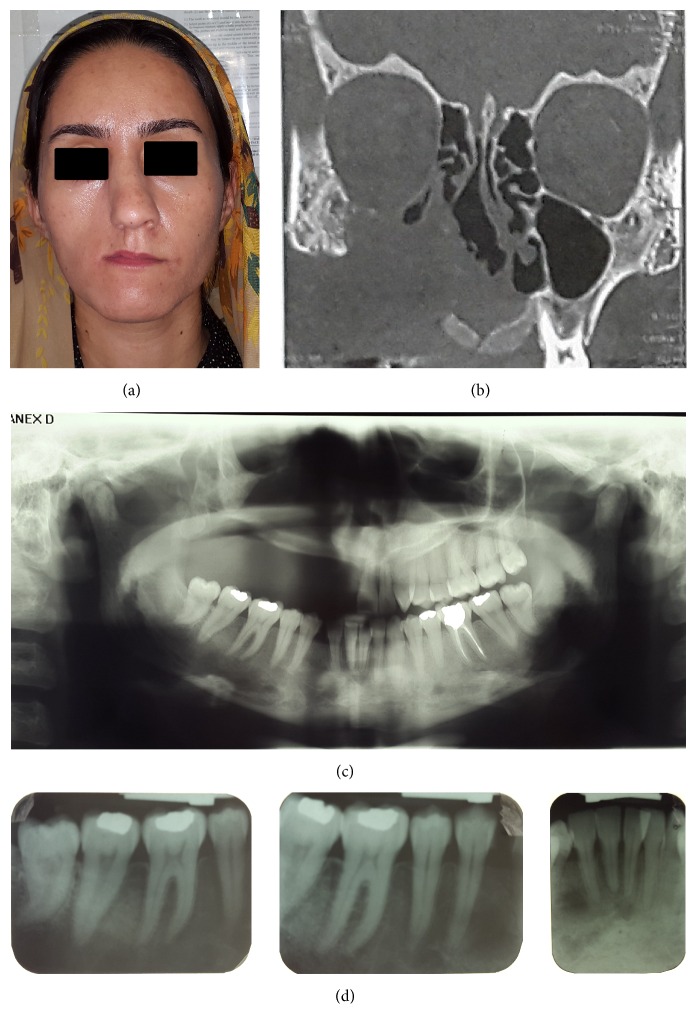
Postoperative course (18 months after surgery). (a) Extraoral frontal photography. (b) CT scan coronal axis. (c) Panoramic view. (d) Periapical views.

The patient's postoperative course was uneventful. There was no recurrence after 18 months of follow-up. She visited with the aim of reconstructing the maxilla, after 18-month recall (Figure 6(a)).

Spiral CT of the coronal axis showed a large defect of the right maxillary alveolar process. Areas of the right maxillary antrum, lower portion of the nasal cavity, and floor of the orbit were replaced with soft tissue and the glob structure was normal (Figure 6(b)). In radiographic evaluation of the mandibular region, the lesion area was healed and bone scar was seen (Figures 6(c) and 6(d)).

In the time of follow-up, the patient was not reconstructed and unfortunately after 2.5 years, she had not survived due to cardiac ischemic attack.

## 3. Discussion

ABC constitutes 0.5% of all types of jaw cysts and 1.5% of nonodontogenic cysts [10, 11]. Mandible-to-maxilla incidence ratio is 3 : 2 and the ramus and molar areas are the most affected sites [10, 12]. In Kalantar Motamedi et al.'s study, 58% of ABCs occurred in the posterior segment [7]. Mean age occurrence is 20 years. There is no gender predilection [2].

It is considered that the pathogenesis of ABC is hemodynamic alteration or an arteriovenous malformation [13] and its etiology includes bone trauma, intramedullary hematoma, and vascular origin (local hemodynamic disturbances like arteriovenous shunts or malformations). Increase in intraosseous venous pressure due to vascular malformation can lead to expansion of the vascular tissue bed, and, consequently, bone resorption, cyst formation, and cystic appearance in the radiographies are seen [14]. Also, chromosomal alterations of 17p11-13 and 16q22 segments are suggested [6]. One theory classifies ABC as a primary, congenital, or secondary lesion [15]. When it is a secondary phenomenon, it occurs due to microcyst from the cellular oedema in preexisting bone lesion such as ossifying and cementifying fibroma, fibrous dysplasia, and central giant cell granuloma [8].

Giant cell granuloma, ameloblastoma, cherubism, and sarcomas may be proposed in the differential diagnosis. There are internal granular septa in ABCs and giant cell granuloma, which causes similar radiographic appearance of both lesions; however, ABC had more expansion and more prevalence in the posterior of mandible. Also, ameloblastoma may be considered, but this lesion usually occurs in older patients. ABCs may be similar to cherubism, due to giant-cell-like feature, but cherubism is a multifocal bilateral disease.

In the cases with a rapid expansive and destructive clinical feature, it may be misdiagnosed as the soft tissue malignancies like sarcomas. Lee et al. introduced a case report of ABC with clinical and radiological doubt of telangiectatic osteosarcoma; they emphasize that it should be included in the differential diagnosis, especially in the aggressive and destructive nature, and invade the soft tissue. Telangiectatic osteosarcoma is histologically confirmed by space, often blood-filled, and separated via septa containing highly malignant cells [12, 16].

The final result for ABC diagnosis is according to histologic findings. CT scan is used for diagnosis of extension of the lesion [12].

However, this case had secondary hyperparathyroidism and the first possible diagnosis for jaw lesions was Brown tumor and central giant cell granuloma.

According to Verma et al.'s study, from 120 cases of maxillary ABC reports, only 7 cases of primary ABC have been reported until date [2]. Also histopathology of this case was primary ABC.

In Kalantar Motamedi et al.'s study, from 120 cases of maxillofacial ABC, only one case had concurrent mandibular and maxillary ABC [7]. Here we compare some case reports with the present case by age, gender, site of orofacial involvement, duration, and their clinical presentation (Table 2).

Age of cases with maxillary involvement in reports of Tang et al. [17], Sheth et al. [11], Arora et al. [1], and Verma et al. [2] was 17, 8, 6, and 8 years, respectively, whereas in our case patient's age was 27 years. None of the reports had bimaxillary ABC involvement and Arora et al.'s [1] report had concurrent maxillary ABC with central giant cell granuloma of mandible. All case reports cause swelling in clinic, whereas, in our case, ABC of mandible had no clinical presentation. Minimal duration of lesions in reports was 1 month in Behal's [9] report and maximal duration was 6 months, according to Arora et al. [1] and Bharadwaj et al. [4], similar to our case.

Radiographically, ABC may present marked expansion, unilocular or multilocular radiolucency with defined border, thinning of the cortical borders, and honeycomb or soap bubble appearance [2, 4, 7, 9]. The cause of various multiple radiographic appearance of ABC refers to its histopathologic feature [2]. More destructive and less defined lesions as well as rapidly progressive lesions have a vascular type and lesions with less destructive and small localized behavior have a solid feature. The mixed form shows properties of two previous variants [9].

Choice of treatment is enucleation with curettage [17]. Documented options of treatment are curettage, enucleating, embolization, block resection, and systemic calcitonin. There are reports on the self-healing of lesions [10]. The recurrence rate of lesion within the first year after treatment is high and varies [3, 17]. The recurrence rate after resection is less than curettage [9]. Recurrence rate of simple curettage varies from 21% to 50% and recurrence in radical methods varies from 11% to 25% [2]. In the present case report, there was no evidence of recurrence after 18 months of follow-up.

Maxillofacial aneurismal bone cyst and concurrent involvement of the upper and lower jaws is rare. Histopathology evaluation revealed primary ABC. Further information about ethiopathogenesis and management of this entity is necessary.

## Figures and Tables

**Table 1:** Laboratory tests of patients showing abnormalities.

	Test	Result	Unit	Reference range
Blood chemistry	Calcium	8/3	mg/dl	8/6–10/3
	Phosphate	8/2	mg/dl	Adult: 2/5–4/5 Child: 4–7
	Albumin	4/6	gr/dl	3/5–5/2

Endocrinology	PTH intact [ECL]	2037	pg/ml	15–65

Special biochemistry	25OH-vitamin D total [ECL]	66/21	ng/ml	20–32 Desirable: >30 Toxic level: >100

**Table 2:** Summary data about age, gender, site of orofacial involvement, duration, and clinical presentation of ABC in case reports.

Author's name	Age	Gender	Site of orofacial involvement	Duration	Clinical presentation
Present case	27	Female	Maxilla and mandible	6 months	Maxilla: expansion and tooth displacement, intraoral mass in buccal sulcus and palatal Mandible: without clinical presentation
Capote-Moreno et al. [3]	29	Male	Mandible	2 months	Swelling of left mandibular angle
Tang et al. [17]	17	Male	Maxilla	4 months	Swelling of left cheek and loosening of left maxillary teeth and left nasal obstruction Intraoral mass in buccal sulcus and palatal
Sheth et al. [11]	8	Female	Maxilla	1.5 months	Swelling of right nasomaxillary region, obliteration of labial sulcus, and central and lateral teeth displacement
Behal [9]	40	Female	Mandible	1 month	Swelling of right angle of mandible
Arora et al. [1]	6	Male	Maxilla [ABC] Mandible [CGCG]	6 months	Swelling in left cheek and mandible
Verma et al. [2]	8	Male	Maxilla	2.5 months	Swelling of right cheek, protrusion of right eye, bulge in right side of hard palate, and ectopic molar tooth inside of lesion
Bharadwaj et al. [4]	12	Female	Mandible	6 months	Asymmetry and swelling of mandible and displacement of right molar and premolar
